# A 10-Year Follow-Up of an Approach to Restore a Case of Extreme Erosive Tooth Wear

**DOI:** 10.3390/dj13060259

**Published:** 2025-06-10

**Authors:** Davide Foschi, Andrea Abate, Francesca Vailati, Ignazio Loi, Cinzia Maspero, Valentina Lanteri

**Affiliations:** 1 Private Practice, 40100 Bologna, Italy; dav.foschi@gmail.com; 2 Department of Sciences Integrated Surgical and Diagnostic, University of Genova, 16126 Genova, Italy; 3 Private Practice, Department of Fixed Prosthodontics and Biomaterial, University Clinic for Dental Medicine of Geneva, 1205 Geneva, Switzerland; francesca.vailati888@gmail.com; 4 Private Practice, 09125 Cagliari, Italy; loi.ig@tiscali.it; 5 Department of Biomedical Surgical and Dental Sciences, University of Milan, 20142 Milan, Italy; 6 Fondazione IRCCS Cà Granda, Ospedale Maggiore Policlinico, 20142 Milan, Italy; 7 Surgical, Medical and Dental Department, University of Modena and Reggio Emilia, 41124 Modena, Italy; valentina.lanteri@unimore.it

**Keywords:** speed-up therapy, set-up, mock-up, smile aesthetics, orthodontics, full-mouth rehabilitation

## Abstract

**Background:** In recent years, thanks to the improvement of adhesive techniques, patients affected by tooth wear, related to erosion and/or parafunctional habits, can undergo restoration by adding only what has been lost of their dentition (additive approach). However, since not all clinicians are convinced that dental rehabilitation should be proposed in the early stages of exposed dentin, several treatments are often postponed. It is important to emphasize that, in the early stages, the clinical approach should remain conservative, focusing on dietary counseling, the modification of harmful habits, fluoride application, and risk factor management. Only when these preventive and non-invasive strategies prove insufficient, and the condition continues to progress, should invasive restorative treatments be considered. Unfortunately, epidemiological studies are reporting an increase in the number of young patients affected by erosive tooth wear, and not intercepting these cases earlier could lead to a severe degradation of the affected dentition. In addition, parafunctional habits are also becoming more frequent among patients. The combination of erosion and attrition can be very destructive, and may progress rapidly once dentin is exposed and the risk factors remain unaddressed. The aim of this report was to present a conservative full-mouth rehabilitation approach for severe erosive lesions and to provide a 10-year follow-up assessing the biological, functional, and esthetic outcomes. **Methods:** In this article, the postponed restorative treatment of a patient, suffering from severe tooth wear, is illustrated. The patient had sought dental treatment in the past; however, due to the already very compromised dentition, a conventional but very aggressive treatment was proposed and refused. Four years later, when the patient finally accepted an alternative conservative therapy, the tooth degradation was very severe, especially at the level of the maxillary anterior teeth. The combination of three different approaches, Speed-Up Therapy, BOPT (Biologically-Oriented Preparation Technique), and the 3 Step Technique, however, improved the capacity to successfully complete the difficult therapeutic task. **Results:** The biological goals (maintenance of the pulp vitality of all of the teeth and the minimal removal of healthy tooth structure) were accomplished, relying only on adhesive techniques. **Conclusions:** The overall treatment was very comfortable for the patient and less complicated for the clinician. At 10-year follow-up, biological, functional, and esthetic success was still confirmed.

## 1. Introduction

In the past decade, clinicians have become more attentive in detecting dental erosion and tooth wear in general.

**Figure 1:**
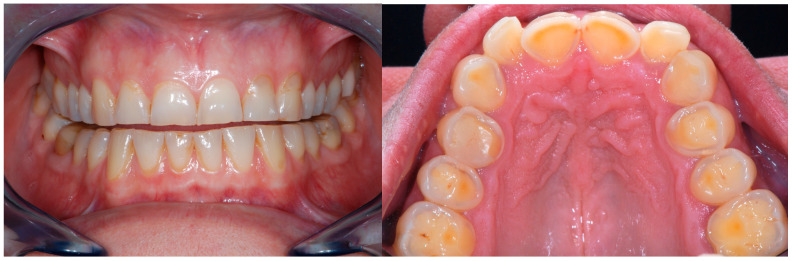
Example of two patients affected by generalized tooth wear.

This raised awareness may also be related to patients’ increased demand for repairing fragile teeth, mainly for esthetic reasons (e.g., the incisal edges of the maxillary anterior teeth) [1,2]. In addition to erosion, in Western countries, an increase in parafunctional habits (e.g., grinding or clenching) has become more noticeable among patients [3,4]. The latter could be greatly implicated in the process of tooth degradation, especially when combined with dental erosion (Figure 1) [5].

Tooth wear is defined as the progressive, non-carious loss of dental hard tissue due to mechanical and/or chemical processes. It is considered a multifactorial condition, typically resulting from the combined effects of friction (attrition), stress (abfraction), and chemical dissolution (erosion). Given the dynamic and complex nature of the oral environment, it is highly unlikely that only one etiological factor acts in isolation. Instead, these mechanisms often coexist and interact over time [5].

A clear distinction should be made between dental erosion and erosive tooth wear. Dental erosion refers specifically to the loss of tooth structure due to acid exposure not involving bacterial activity, often caused by dietary acids or intrinsic factors such as gastroesophageal reflux. Erosive tooth wear, on the other hand, describes the clinical manifestation of tooth surface loss when erosion occurs in combination with other mechanical forces, such as attrition or abrasion.

Attrition is the mechanical wear of tooth surfaces caused by direct tooth-to-tooth contact, commonly seen in patients with parafunctional habits such as bruxism or clenching. These habits are increasingly prevalent and represent a significant contributing factor to tooth wear [3].

The prevalence of tooth wear is increasing, particularly among adolescents and young adults, largely due to modern dietary habits (e.g., acidic beverages), increased stress levels, and parafunctional behaviors. Risk factors include the frequent intake of acidic foods and drinks, gastric reflux, xerostomia, bruxism, incorrect toothbrushing techniques, and systemic conditions affecting salivary flow or pH regulation [3].

Several negative consequences may derive from the combination of erosion and attrition, starting from the thinning and loss of the enamel with progressive exposure of dentin. The loss of the tooth morphology, which originates, has an impact not only on the esthetic of the smile, but also on the stability of the occlusion (functional problems).

The esthetic impairment involves both changes in the shape of the affected teeth, by weakening and shortening incisal edges and cusps (e.g., reverse smile line), and color change [6,7,8]. The thinning of the enamel, in fact, may lead to a more yellowish dentition, which could worsen, if the exposed dentin becomes discolored. The functional damage is, on the other hand, related to the instability of the occlusion with supraerupted teeth, the worsening of deep bite, an accentuated curve of Spee, and a loss of vertical dimension of occlusion (VDO). Finally, potential problems related to biological damage (from dentin hypersensitivity to pulp necrosis) should convince clinicians not to postpone dental therapy.

Thanks to adhesive techniques, the vertical dimension and the correct occlusal morphology can be restored by minimally invasive techniques (direct or indirect additive approaches) that allow for significant saving of biological tissue [9]. In many cases, preliminary orthodontic treatment can help to resolve various types of dental incongruities and correct irregularities in arch shape and occlusal plane or loss of vertical dimension, reducing the need for compromise solutions that could have negative repercussions on prosthetic work prognosis and customer satisfaction [10,11,12,13,14]. Moreover, a study on the treatment of extensive erosion damage with a consistent follow-up has, according to the current scientific literature, not been published before.

Therefore, the aim of the present report was to illustrate a new conservative integrated method for the rehabilitation of the full mouth, for the treatment of severe lesions of erosive origin, and to report a 10-year follow-up of the patient, evaluating the biological, functional, and esthetic parameters.

## 2. Case Report

A 48-year-old Caucasian man presented to the private practice of one of the authors. His chief complaint was that his maxillary anterior teeth were not visible upon smiling. He did not report any impairments of his chewing ability, which was also confirmed by the absence of signs or symptoms of temporo-mandibular joint disorders during the clinical evaluation. While there was no complaint about function, the esthetic of the patient’s smile was very compromised. It was in fact very noticeable that the tooth exposure was minimal to none, even during forced smiling.

**Figure 2:**
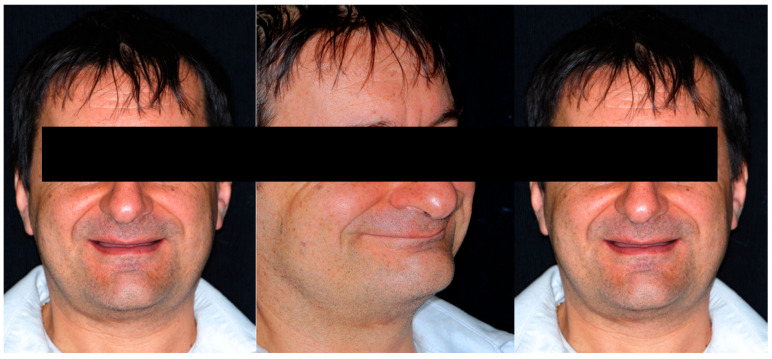
Patient’s profile and smile during the first visit. Due to the tight lips and the very damaged teeth, it was impossible for the patient to show his teeth upon smiling. Note the visible collapse of the lower third of the face when the teeth were in contact.

**Figure 3:**
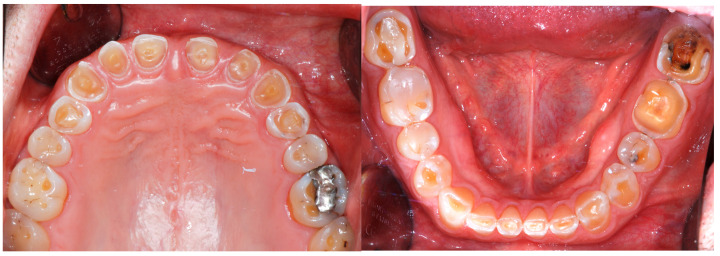
Occlusal view showing the severe loss of tooth structure, especially at the level of the maxillary anterior teeth and the mandibular left molars.

Concerning the aesthetic analysis, the patient exhibited a brachycephalic facial biotype, with a visible collapse of the lower third of the face. The maxillae were biretusive, as was the position of both lips. Due to the tight lips and the very damaged teeth, it was impossible for the patient to show his teeth upon smiling. Note the visible collapse of the lower third of the face when the teeth were in contact, contributing to the aging of his aspect (Figure 2 and Figure 3).

During the intraoral examination, the generalized loss of tooth structure was very noticeable. Since a diagnosis of erosive tooth wear was immediately made, the clinician tried to find the origin of the excessive presence of acids in the mouth. The patient, however, denied any causes of extrinsic dental erosion, such as excessive consumption of acidic foods and or beverages.

To evaluate a possible intrinsic origin (e.g., gastroesophageal reflux), it was proposed to carry out a gastroenterological investigation [15]. The patient, as often it happens, did not follow through. Moreover, we assessed the presence of bruxism and musculoskeletal issues through a detailed clinical examination and anamnesis. The patient was found to exhibit clenching and had a highly developed masticatory musculature. Additionally, the patient reported episodes of nocturnal bruxism, which contributed to the extensive dental tissue loss observed. From a psychological perspective, no significant factors influencing bruxism or clenching were identified. The patient did not report symptoms of stress, anxiety, or other psychological conditions that could have contributed to the parafunctional activity. Therefore, psychological influences were not considered a determining factor in this case.

Since the authors believe that dental treatment for patients affected by erosive tooth wear should start as soon as possible to protect the remaining tooth structure, the dental rehabilitation began (Figure 3).

**Figure 4:**
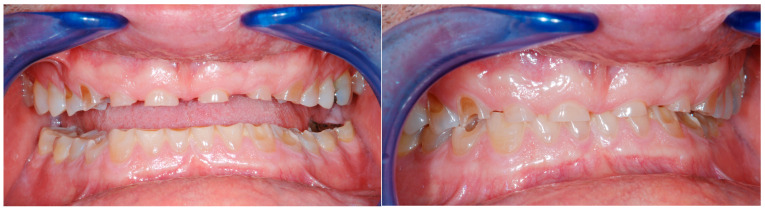
Intraoral view. Despite the generalized conspicuous loss of tooth structure, all the teeth (except one premolar) were still vital, confirming the slow progression of the disease. Note the deep bite and the very tight lips, which made it difficult to take intraoral pictures.

The periodontal status on the other hand was remarkable, with good oral hygiene. No bleeding upon probing, an overall probing depth less than 3 mm, and no tooth mobility. The gingival biotype was thick (Figure 4). At the initial consultation, the patient reported brushing twice daily using a medium-bristled manual toothbrush and a whitening toothpaste with abrasive particles. Although the patient maintained regular oral hygiene, the combination of abrasive toothpaste and a vigorous brushing technique may have contributed to the progression of erosive tooth wear (ETW), particularly in the presence of already demineralized surfaces due to acid exposure.

**Figure 5:**
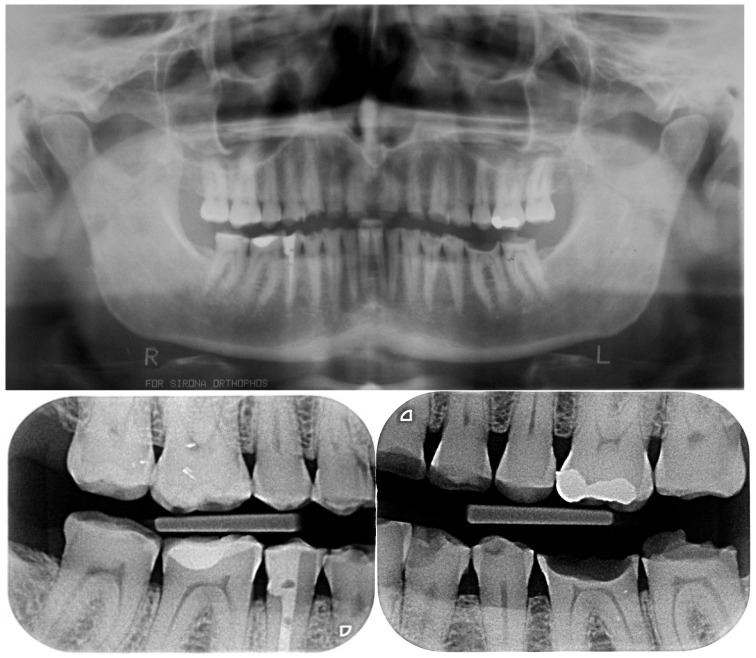

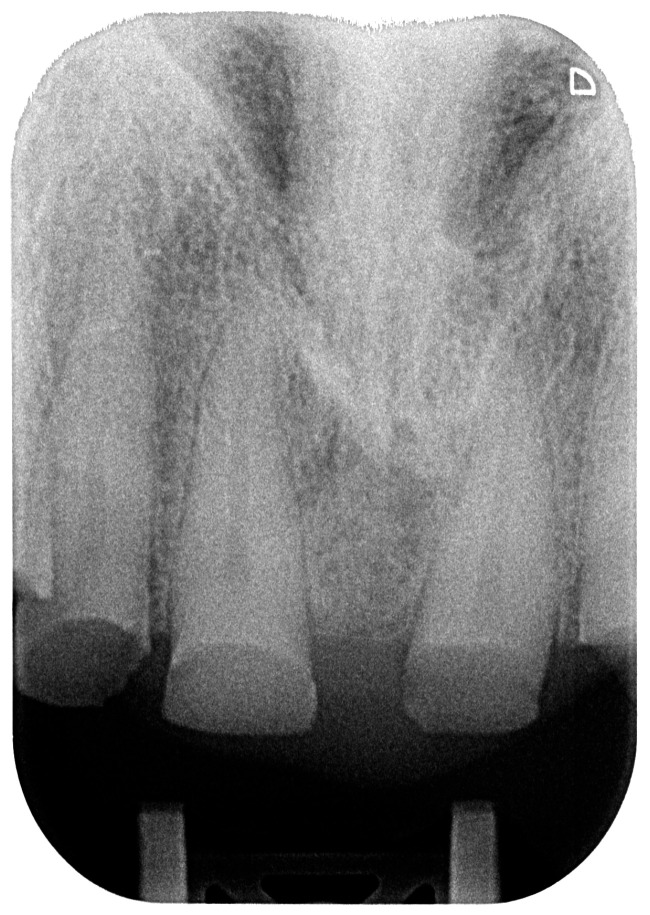
Initial radiographic status.

Despite the severe wear, the damaged teeth still responded positively to the vitality test except the right mandibular premolar, previously endodontically treated (Figure 5).

The patient was in possession of accurate intraoral photographic documentation, obtained during a previous dental consultation. The patient had in fact sought dental treatment four years before, but the cost and invasiveness of the proposed treatment (elective endodontic therapy, crown lengthening, and crowns on every tooth) had pushed him to refuse such a therapy [16,17].

**Figure 6:**
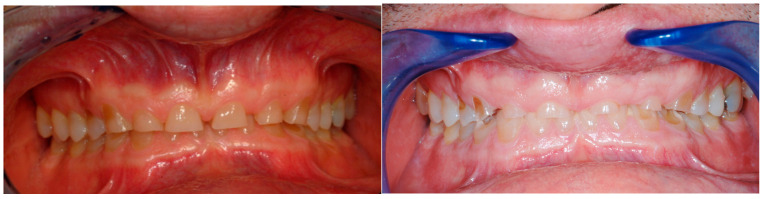
Comparison between the dentition of the patient photographed by another clinician and the dental degradation which occurred by leaving the patient without any dental therapy for four years. The severity of the tooth wear supports the authors belief in the early interception of patients affected by tooth wear.

Photographic records were taken during the patient’s initial consultation, at which time the clinical condition already required restorative intervention. However, the patient declined treatment at that stage, and no follow-up visits occurred until several years later, when the patient returned with a significantly worsened clinical situation. Although the progression was not documented through regular follow-up, the comparison between the initial and subsequent photographs provides clear visual evidence of the deterioration over time in the absence of intervention (Figure 6).

Since one of the objectives of the therapy would have been to avoid any additional removal of healthy tooth structure in such a compromised dentition, additive therapy was advocated. A full-mouth adhesive rehabilitation at an increased vertical dimension of occlusion (VDO) was proposed to the patient during the first visit, emphasizing the non-invasive approach of the therapy [18]. The patient was very pleased with this prospect. The initial visit was concluded with two alginate impressions and a face bow. The models were mounted in maximum intercuspidal position (MIP) and the clinician started the evaluation of the case [19].

Considering the severe loss of tooth structure, the increase in VDO needed to reestablish the correct dental forms would have been conspicuous, while the dentition presented limited tooth surfaces available to bond, especially at the level of the maxillary anterior teeth.

Thus, questions were arising not only about the adaptability of the patient to the new occlusion, but also the resistance of the bond of the restorations. Since it was excluded to devitalize the anterior teeth for restorative proposes (e.g., elective endodontic therapy), the experimental dental treatment was deeply discussed with the patient. After obtaining the patient’s informed consent, the project of the conservative full-mouth rehabilitation started.

Guidelines on how to start a full-mouth additive rehabilitation have been developed by the 3 Step Technique, to help clinicians and laboratory technicians in better planning difficult cases. The 3 Step Technique, developed in 2005, is a minimally invasive protocol for rehabilitating worn or eroded teeth. It follows an additive and adhesive approach, preserving natural tooth structure without the need for drilling or root canal treatments. The technique includes three main phases: (1) Functional and esthetic mock-up—A digital scan is used to create a resin mock-up, allowing evaluation and adjustment of the esthetic and functional outcome; (2) Reconstruction of posterior teeth and increase in vertical dimension of occlusion (VDO)—Premolars and molars are restored with composite or lithium disilicate to restore VDO and create space for anterior reconstruction; (3) Anterior reconstruction—Palatal surfaces of incisors and canines are restored using composite or ceramic veneers.

This technique preserves tooth structure, reduces discomfort, and provides a predictable, reversible outcome [20,21,22].

Since doubts were present on the amount of VDO required, a different approach was preferred, the Speed-Up Therapy (full-mouth therapeutic mock-up). The classic protocol of the 3 Step Technique was in fact modified with the aim of restoring the entire dentition, anterior and posterior teeth, during the same visit by delivering a therapeutic mock-up, instead of the white therapeutic bite of the 3 Step Technique [9,23,24,25].

The clinician felt more confident rehabilitating the patient with a new posterior support at the established VDO. A mock-up was the ideal solution to test the new occlusion in a reversible way.

This extended mock-up would be bonded on the tooth surfaces of all the patient’s teeth, allowing a more global evaluation of the function and esthetic outcome of the planned rehabilitation (therapeutic mock-up).

**Figure 7:**
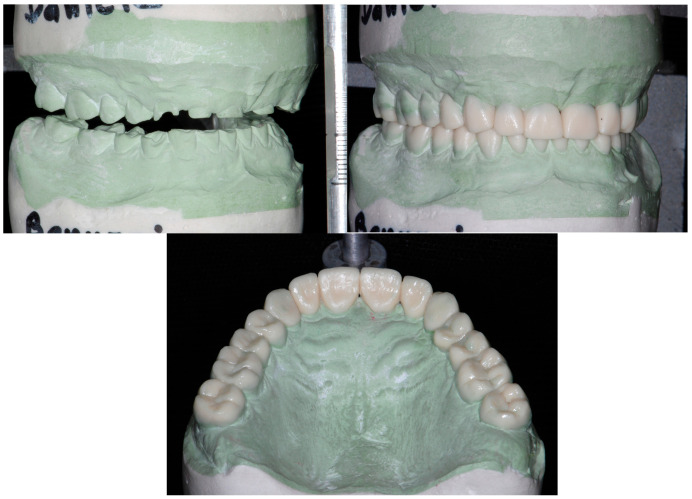
Instead of following the partial wax-up of the 3 Step Technique, the laboratory technician was instructed to complete a full-mouth wax-up. The increase in VDO was arbitrarily decided on the articulator looking at the restorative needs of the specific case.

Following the Speed-Up Therapy, the first diagnostic wax-up included all of the teeth, which were waxed-up to the final proposed shape. The increase in VDO was 8 mm at the level of the pin of the articulator. This increase was arbitrarily decided on the articulator, considering the space necessary to reconstruct the maxillary anterior teeth with anatomically acceptable forms. The restorative requirements, which guided the diagnostic wax-up, were the position of the incisal edges in harmony with the esthetic occlusal plane, a less accentuated curve of Spee, and a reduced vertical overbite (deep bite) (Figure 7).

Each waxed-up arch was then duplicated with extra-rigid silicone (Platinum 95-Zhermack 95 shore) to fabricate two mock-up keys. The patient was then scheduled for a 2 h appointment.

Regarding the maxillary anterior quadrant, there was a fear that the minimal tooth structure left was not enough to hold the mock-up in place. Consequently, before the fabrication of the mock-up, these teeth were reconstructed with a direct composite core build-up, without any posts (donut-shaped dentin preparation). The tooth preparation consisted only of removing the most superficial and sclerotic layers of the exposed dentin with a diamond bur.

To confirm the minimal invasiveness of the treatment and the sclerotic aspect of the dentin, anesthesia was not necessary to complete this task.

**Figure 8:**
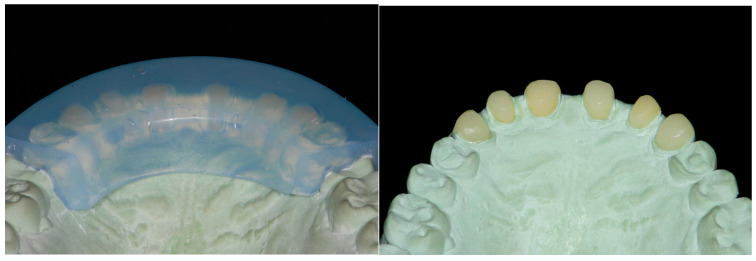

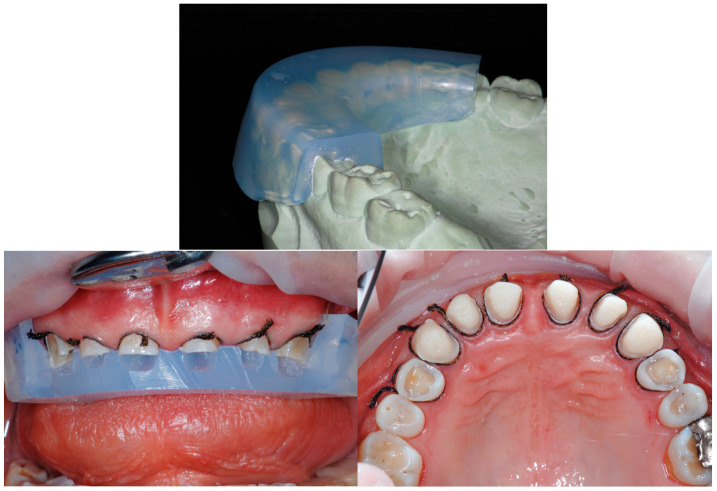
The maxillary anterior teeth were not devitalized but restored with a core build-up in composite fabricated directly in the mouth by means of a transparent key.

**Figure 9:**
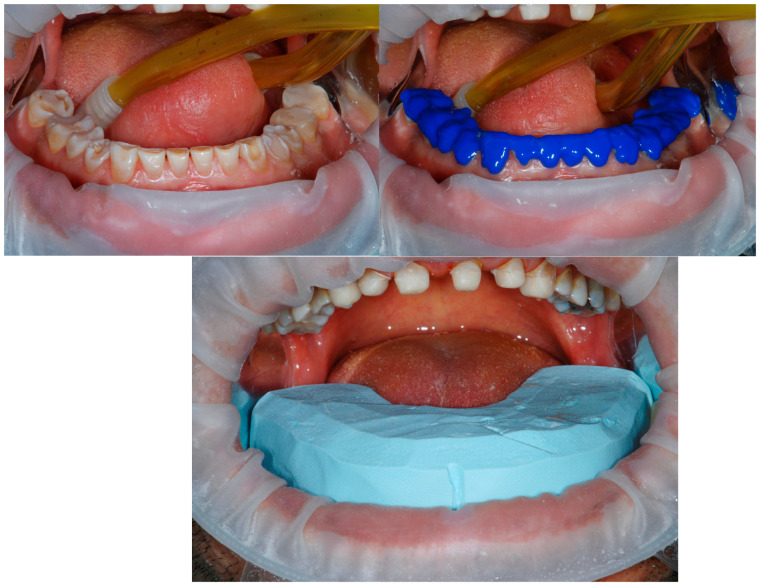
Following the Speed-Up Therapy, the mandibular arch was isolated and orthophosphoric acid applied on all the surfaces to retain the therapeutic mock-up, fabricated directly in the mouth by means of a silicon key.

To help in their reconstruction, the shape of these cores had been already designed with a wax-up and duplicated with a transparent key (Figure 8 and Figure 9).

After completing the build-up of these teeth, the treatment progressed with the fabrication of the therapeutic full-mouth mock-up.

The keys were tried first in the mouth, since unfortunately one of the inconveniences of a full-mouth wax-up is that the keys for the mock-up do not have any dental stops, and they must rely only on the soft tissues. To try to overcome this problem, the diagnostic wax-ups had been already modified before duplication, by exposing the most cervical areas of the waxed-up teeth. In addition, since the mock-up would be left in place for several months, the clinician preferred improving its strength by being better attached to the teeth. Thus, the tooth surfaces were etched with 37% orthophosphoric acid for 30 s. A layer of adhesive was also applied and polymerized (Optibond FL, Kerr, Kloten, Switzerland).

**Figure 10:**
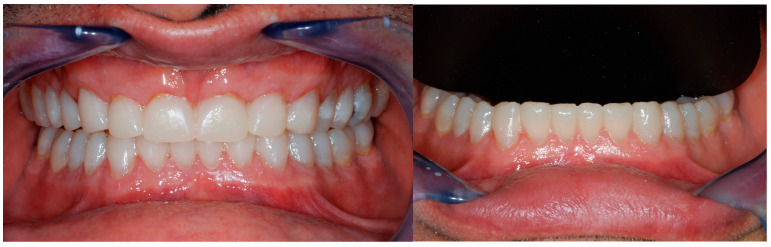
Mandibular arch restored with the therapeutic mock-up and view of both arches immediately after removing the silicon keys. Minimal excesses were present, and the occlusion required very few adjustments, thanks to the modification of the wax-up at the cervical level and the rigidity of the keys.

Finally, the silicone keys, one at the time, were filled with the provisional composite (TELIO C&B Chairside, Ivoclar, Schaan, Liechtenstein) and positioned in the mouth. At their removal, the mock-up remained pressed on the patient’s dentition, recreating the laboratory project (Figure 9 and Figure 10).

After eliminating the excess, the clinician had the possibility to evaluate with the patient at the same time the three parameters fundamental for full-mouth rehabilitation: the incisal edges’ position, the occlusal plane, and the increase in VDO.

Since the patient was not anesthetized, he was fully cooperative to evaluate his new occlusion.

The goal of the occlusal adjustment was to achieve a stable static occlusion, one contact point for each tooth, a group guide, and a comfortable dynamic occlusion (e.g., group function with the same masticatory functional angles of Planas), which would allow the patient to chew comfortably in both posterior sextants [26,27,28].

**Figure 11:**
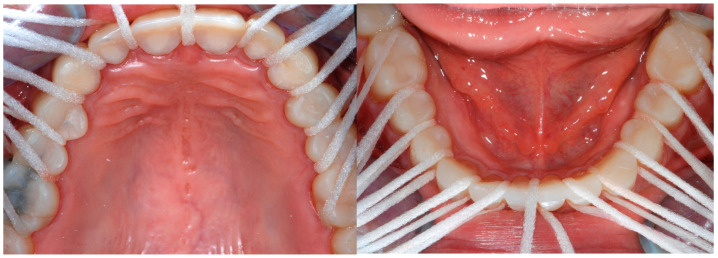
Therapeutic mock-up and super floss. This picture shows the possibility for each gingival embrasure to be accessible and cleansable.

The importance of oral hygiene was stressed, since even though all the gingival embrasures were kept open, the interproximal contacts were closed, and the mock-up had a rough surface more prone to plaque accumulation (Figure 11).

The fabrication of the two mock-ups and the occlusal adjustments took 2 h, and the patient was then rescheduled for a follow-up visit after 3 weeks. One of the main concerns was the non-acceptance of the new increased VDO with the onset of discomfort at the level of the temporo-mandibular joints or parafunctional habits. At the 3-weeks follow-up, the patient was instead very comfortable with the new occlusion. Two alginate impressions were taken with a face bow, and the casts were mounted in the semi-adjustable articulators and analyzed. Even though the patient was already satisfied with the proposed rehabilitation, from a functional and esthetic point of view, an additional increase in the VDO was considered necessary. The decision to increase the VDO was related to esthetic needs to deliver anterior restorations with a more harmonious shape, and restorative needs to avoid the creation of a deep bite, while lengthening the anterior teeth.

The decision was made to increase the VDO by an additional 2 mm at the articulator’s pin (total increase 10 mm) using another mock-up.

Two silicon keys were used to add thickness to the posterior teeth in the mandibular arch to avoid a deep bite.

The new layer of the same product was pressed (after sandblasting and bonding of the composite provisional restoration’s surfaces—Optibond Fl Kerr) on the mock-up without the need to replace the existing provisional restorations.

While the patient was test-driving the new occlusion, the clinician was also planning which type of final restoration to deliver, especially for the anterior maxillary teeth.

Due to the very limited amount of remaining tooth structure, the bilaminar approach of the 3 Step Technique (a palatal and a facial veneer to restore each anterior tooth) was not indicated [29]. It was then decided to restore these teeth with crowns. No crown lengthening or elective endodontic therapy was considered, however.

To save the maximum amount of remaining tooth structure, a crown preparation with a classic margin design (e.g., chamfer or shoulder preparation) was also excluded, as it would have weakened the abutments at their cervical level.

To preserve the ferrule effect, and to close the existing anterior diastema with a very pronounced emergency profile, it was decided to restore these teeth, following the BOP technique, which advocates feather-edge preparation of the abutments [30].

After 3 months of Speed-Up Therapy (therapeutical mock-up), the day of the removal of the mock-up in the anterior segment, the clinician was pleased to see that the cores were holding in place despite the very small surface of retention.

**Figure 12:**
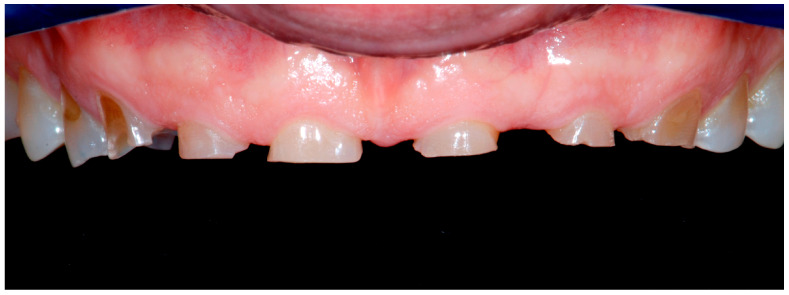

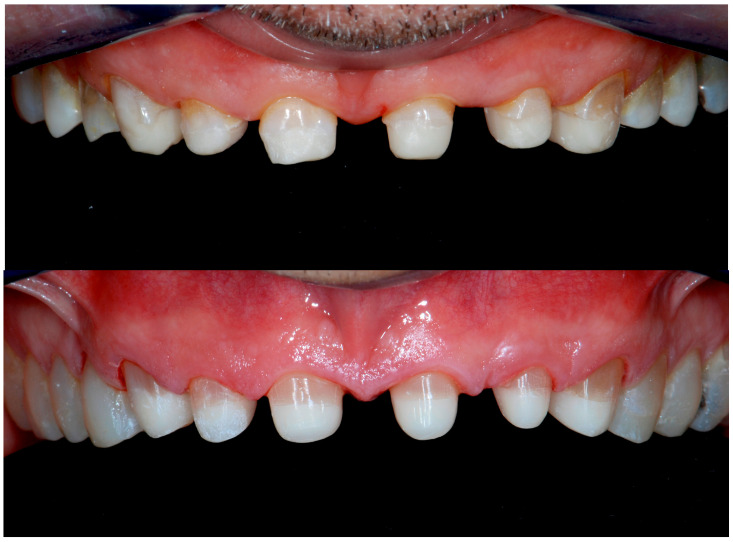
Teeth preparation (initial, abutments, final prep).

Thanks to the knife-edge preparation of the BOP technique, the tooth preparation was limited only to the elimination of minor undercut areas, to the point that anesthesia was not necessary during this procedure (Figure 12).

To prepare the teeth in the meantime, the initial wax-up was used to fabricate a mock-up key for the six maxillary anterior teeth.

**Figure 13:**
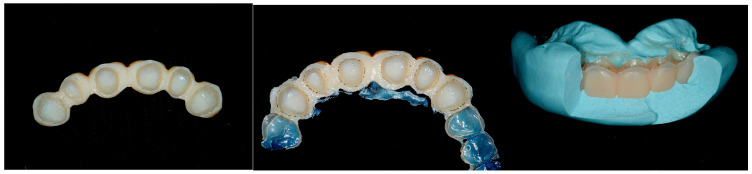
First provisional restoration made with a mock-up key directly in the mouth.

This key was filled with provisional composite (Telio C&B Chairside, Ivoclar) and pressed into the mouth. Since the abutments were covered with glycerin, the polymerized resin remained in the key, and it was refined and polished before being cemented in the mouth with a temporary cement (Temp Bond Kerr) (Figure 13).

**Figure 14:**
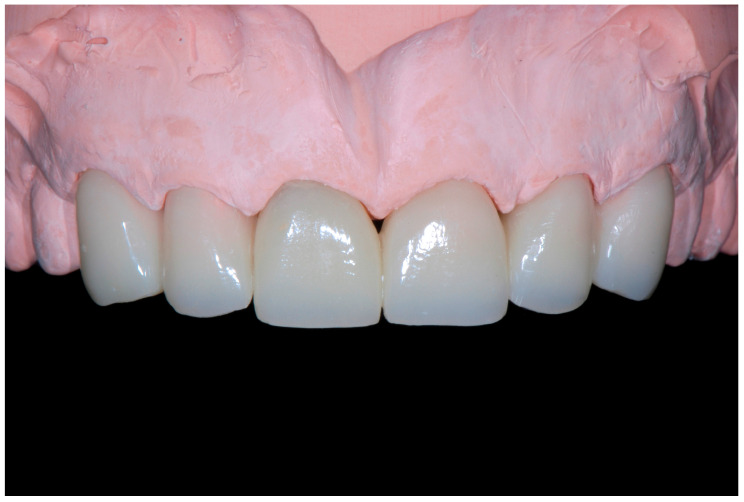
Laboratory-made provisional with BOPT emergency profile.

A second laboratory-made provisional was fabricated, following the guidelines of the BOPT (Biologically-Oriented Preparation Technique) on the emergency profile, where the six teeth were splinted together (Figure 14).

While delivering the new provisional, the clinician used two silicon keys to increase the VDO at the level of the maxillary posterior teeth (+2 mm). The existing mock-up was left in place, but to adhere to the new layer, it was sandblasted, and adhesive was applied and cured (Optibond FL, Kerr, Kloten, Switzerland). The keys were filled with provisional composite resin and pressed onto each sextant. At their removal, the teeth were restored with thicker restorations (Telio C&B Chairside, Ivoclar).

With the new provisional restorations in place, the new occlusion (new increase in VDO, total increase 12 mm at the articulator’s pin) was checked with the patient fully cooperative, since even this time no anesthesia was delivered.

While several months passed by to test the function with the provisional restorations, the clinician started to debate the choice of the dental material for the final restorations. Finally, the material chosen for the posterior teeth was a CAD/CAM hybrid ceramic, consisting of a composite resin matrix enriched with nano-particles of zirconium oxide (Lava Ultimate, 3M, St. Paul, MN, USA). For the anterior crowns and veneers (mandibular teeth), glass ceramic would have been a recommended choice [31].

However, one of the problems in restoring the posterior teeth in composite and the anterior teeth in ceramic is the different rate of wear of the two materials, related not only to the intrinsic characteristics of each material, but also to the different occlusal load.

With time, the risk of more pronounced wear of the posterior support in the composite and an excess overload of the anterior contacts in the ceramic should be anticipated.

In the authors’ opinion, when the posterior teeth are restored in composite, the same material should be selected for the anterior teeth.

Since it was not considered appropriate to deliver posterior restorations in ceramic for this patient, due to the multiple occlusal adjustments potentially necessary to reach his comfort, the choice of ceramic restorations for the anterior teeth was placed aside, even for the antagonistic mandibular veneers.

**Figure 15:**
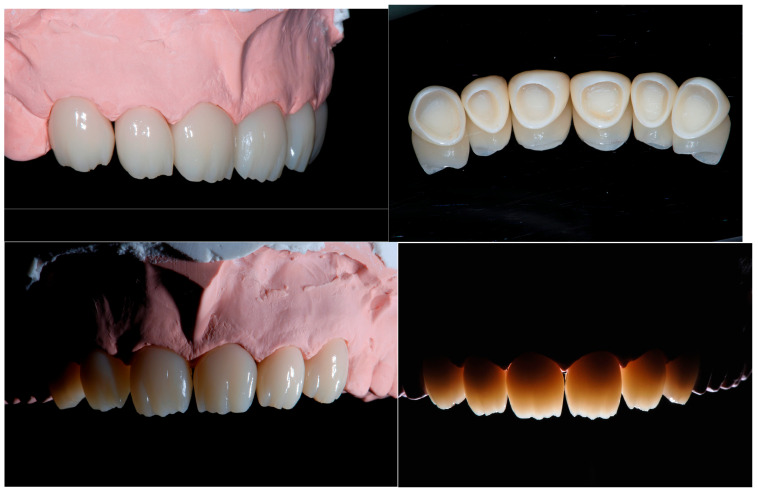
CAD/CAM composite crowns with minimal cutback.

Consequently, the choice for the restorations of all the teeth was CAD/CAM hybrid ceramic (resin nano ceramic). Even though recent doubts have been raised about the clinical validity of CAD/CAM hybrid ceramic crowns, 12 years ago, these warnings were not present, and the choice of the material was dictated by the recreation of a dentition made from the same material with no fear of debonding. In order to obtain a better integration from an esthetic point of view, it was decided to carry out a cutback on the vestibular surfaces of the monolithic restorations (Filtek Supreme, 3M Company, St. Paul, Minnesota, USA). To avoid excessively weakening the core of each crown, the cutback was kept to a minimum (Figure 15).

The day of the cementation of the final crowns, it was possible to appreciate the healthy status of the soft tissues and a good conditioning of the scalloped gingiva.

**Figure 16:**
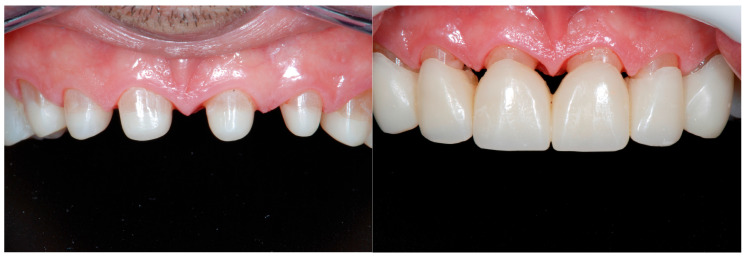
Close view of the removal of the laboratory-made provisional restorations and the abutments ready to receive the final restorations. Note the status of the soft tissue.

The procedure did not require anesthesia (Figure 16).

Restoration conditioning: During the delivery of the crowns, the intaglio surface of the hybrid ceramic restorations was sandblasted with 50-micron particles of aluminum oxide, and the crowns were cleaned in an ultrasonic bath with alcohol. A silane coupling agent (e.g., Monobond Plus, Ivoclar Vivadent) was applied and allowed to react for 60 s, then air-dried. Adhesive resin was placed and not cured (Optibond FL, Kerr, Kloten, Switzerland).

Tooth surface preparation: The teeth were cleaned with pumice and isolated.

The abutments were sandblasted with 27-micron particles (Cojet Sand, 3M, St. Paul, MN, USA) at the level of the composite core build-up.

Enamel margins were selectively etched with 37% phosphoric acid for 30 s, the dentin was etched with 37% orthophosphoric acid for 20 s, then rinsed and gently air-dried to avoid desiccation of dentin. Adhesive resin was placed and not cured (Optibond FL, Kerr, Kloten, Switzerland). 

**Figure 17:**
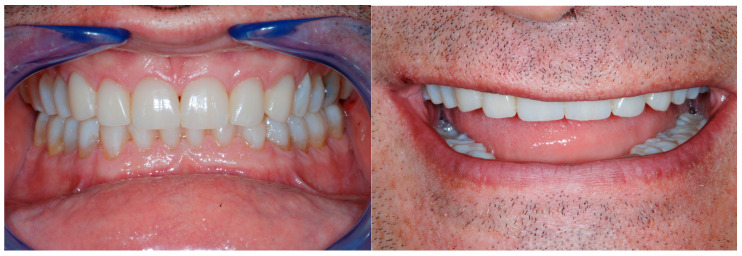
Follow-up at 2 weeks after delivering the final restorations in the premaxilla. The treatment progressed with the replacement of the antagonistic provisional restorations and after with the posterior teeth by quadrant.

**Figure 18:**
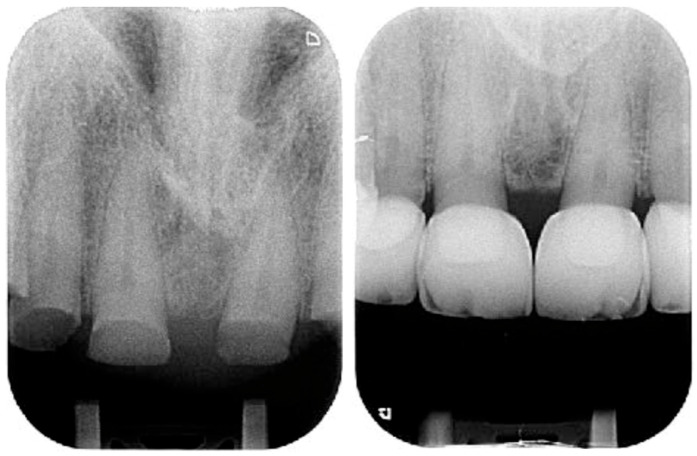
Initial radiographs and after bonding the anterior maxillary final restorations. Due to the different radio opacity, it is possible to see the size of the core build-up and the remaining monolithic CAD/CAM composite with the very little cutback.

An hybrid composite (HFI Enamel Plus Dentin, Micerium, Avegno, Italy) was used as cement (Figure 17 and Figure 18). Excess cement was removed, and each surface was light-cured for 80 s to ensure complete polymerization.

After the delivery of the final maxillary anterior restorations, the rehabilitation progressed with the replacement of the mandibular anterior provisional restorations.

**Figure 19:**
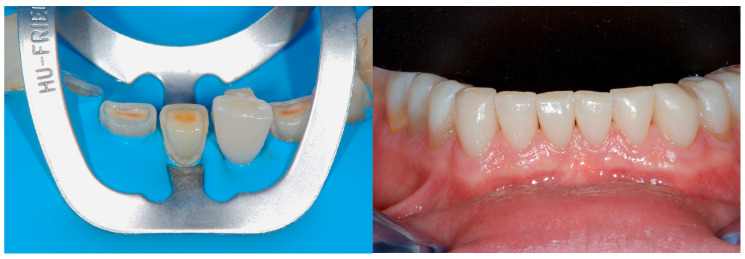
Delivery of the composite facial veneers and follow-up 2 week later. The rehabilitation progressed then with the replacement of the posterior provisional restorations by quadrant.

After carefully removing the temporary restorations, the teeth were only minimally prepared and the exposed dentin sealed before the final impression, following the protocol proposed by Dr. Pascal Magne [32,33,34,35]. After 1 week, the facial hybrid ceramic veneers (Lava ultimate, 3M ESPE, St. Paul, MN, USA) were bonded using a hybrid composite (HFI Enamel Plus Dentin, Micerium, Avegno, Italy), following the same adhesive protocol illustrated for the crowns (Figure 19).

After the delivery of the final anterior restorations, the rehabilitation progressed with the replacement of the posterior provisional restorations, by quadrant.

In private practice, the possibility of segmenting a full-mouth rehabilitation is very important and not only for the comfort of the patient.

**Figure 20:**
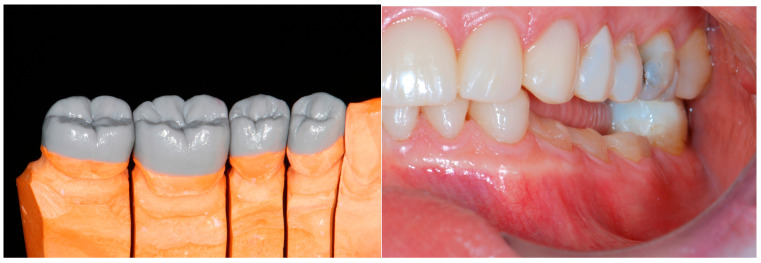
Wax-up for the fabrication of the CAD/CAM restorations.

Each appointment was scheduled for 2 h, during which the provisional restorations were removed, the dentin sealed, and the impression taken. After that, the provisional restoration was fabricated with silicon keys duplicating the dental status before removing the provisional restorations (Figure 20).

A wax-up for the fabrication of the CAD/CAM monolithic composite restorations was created. Note that after the removal of the provisional restorations, there was a conspicuous increase in VDO and a very reduced tooth structure left. These teeth would have been devitalized in the case of crown preparation. Thanks to the adhesive techniques, they kept their vitality.

**Figure 21:**
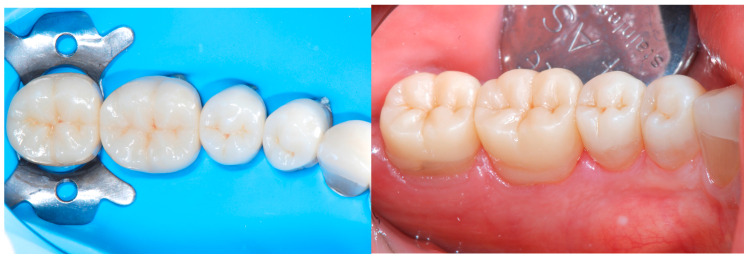
Delivery of the final restoration and 1-month follow-up.

**Figure 22:**
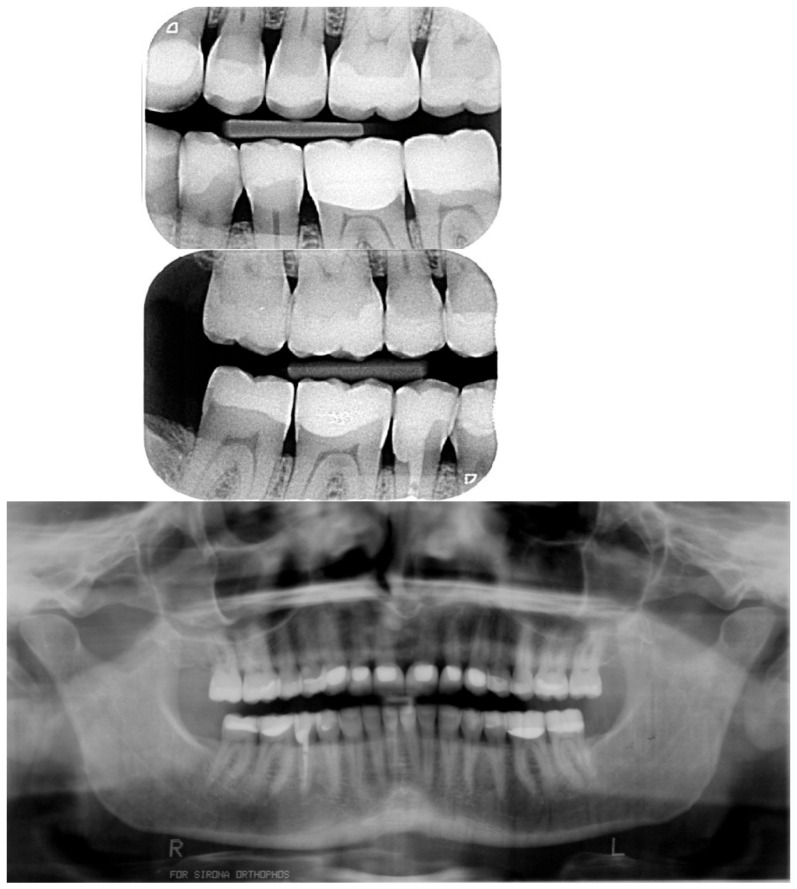
Radiographs after delivery of the restorations. All the teeth kept their vitality.

Figure 20 illustrates the delivery of the final restoration and 1-month follow-up. Since the isolation of the operatory field was mandatory, the restorations were delivered by quadrants, so that the clinician had the possibility to really concentrate on the task of bonding them. Finally, a complete set of radiographs were taken (Figure 21 and Figure 22).

**Figure 23:**
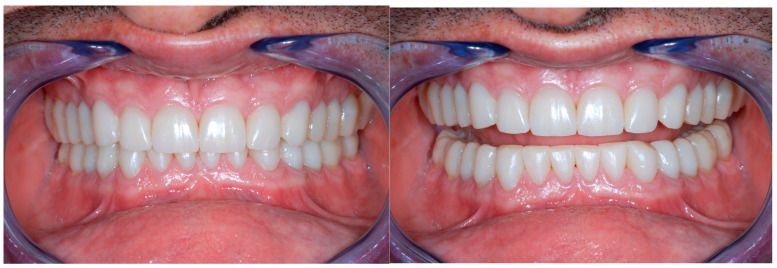
Completion of the full-mouth adhesive rehabilitation.

**Figure 24:**
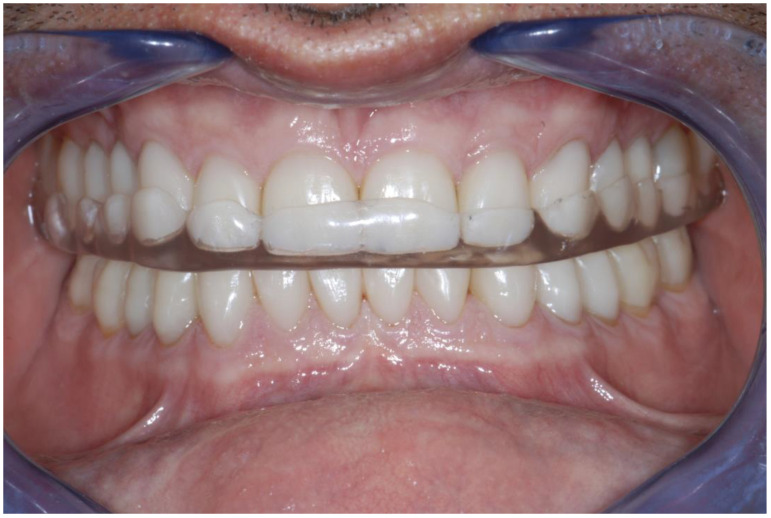
A Michigan occlusal guard was delivered to the patient, in addition to an umbrella bite to re-mineralize the unrestored tooth surfaces (as the erosion risk is still present).

**Figure 25:**
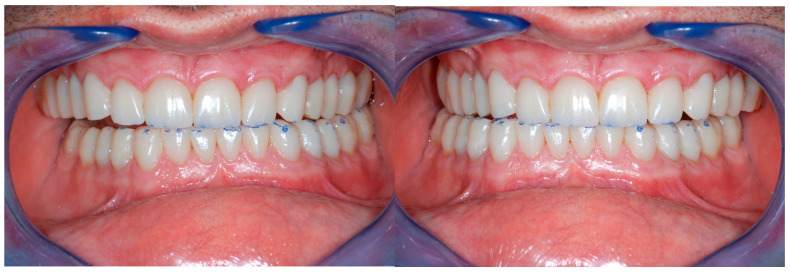

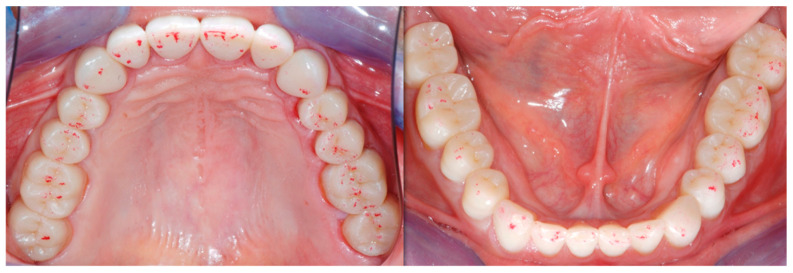
Dynamic occlusal adjustments and occlusal view of the static occlusion.

At the completion of the rehabilitation, an occlusal night guard was delivered to the patient. Since the patient did not accept a visit with the gastroenterologist, the intrinsic origin of the acid was not investigated (Figure 23, Figure 24 and Figure 25). In cases like this, when the risk of the presence of acid is still there, an umbrella bite should be delivered to the patient.

An umbrella bite is a thermoformed bite which extends even more on the gingiva.

The patient is instructed to fill this bite with paste containing fluoride or calcium phosphate (Tooth Mousse GS MI PASTE) to re-mineralize the surfaces not protected by the restorations.

During the lateral excursions, the patient was opening the VDO in a similar manner (similar functional masticatory angles of Planas), which was a good indication that the patient would have chewed with the same comfort on both the side of the mouth.

**Figure 26:**
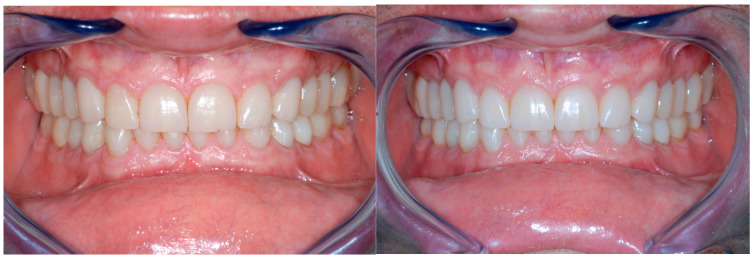
Follow-up at 2 years and at 4 years.

The patient entered into a follow-up program with a structured maintenance phase to ensure the long-term stability of the treatment outcomes. Follow-up visits were scheduled at 1, 3, 6, and 12 months post-treatment. During these visits, clinical evaluations were performed, including occlusal assessment, percussion tests, pulp vitality tests, and radiographic analysis to monitor any signs of relapse, root resorption, or periapical complications. Additionally, patient compliance with oral hygiene instructions and the use of retention devices (if applicable) were reinforced at each visit. This structured follow-up protocol aimed to identify and manage potential complications early, thereby enhancing the long-term success of the treatment. Moreover, a pulp vitality assessment during follow-up was performed to evaluate the health and functionality of the dental pulp after treatment. Initially, a detailed anamnesis was conducted to identify any symptoms of sensitivity or spontaneous pain. Clinical tests were then carried out, including the cold test (using ethyl chloride or CO_2_) to assess the sensory response of the pulp. In the absence of a response to sensitivity tests, percussion and palpation were performed to rule out the presence of periapical lesions. Radiographic follow-up was conducted to monitor any signs of root resorption or pulp necrosis. Regular assessments were scheduled at 1, 3, 6, and 12 months to confirm the stability of the pulp response over time. In addition to the patient’s great satisfaction for the therapy, the restorations were aging without any mechanical failures (e.g., chipping) even at the 6 year follow-up (Figure 26).

**Figure 27:**
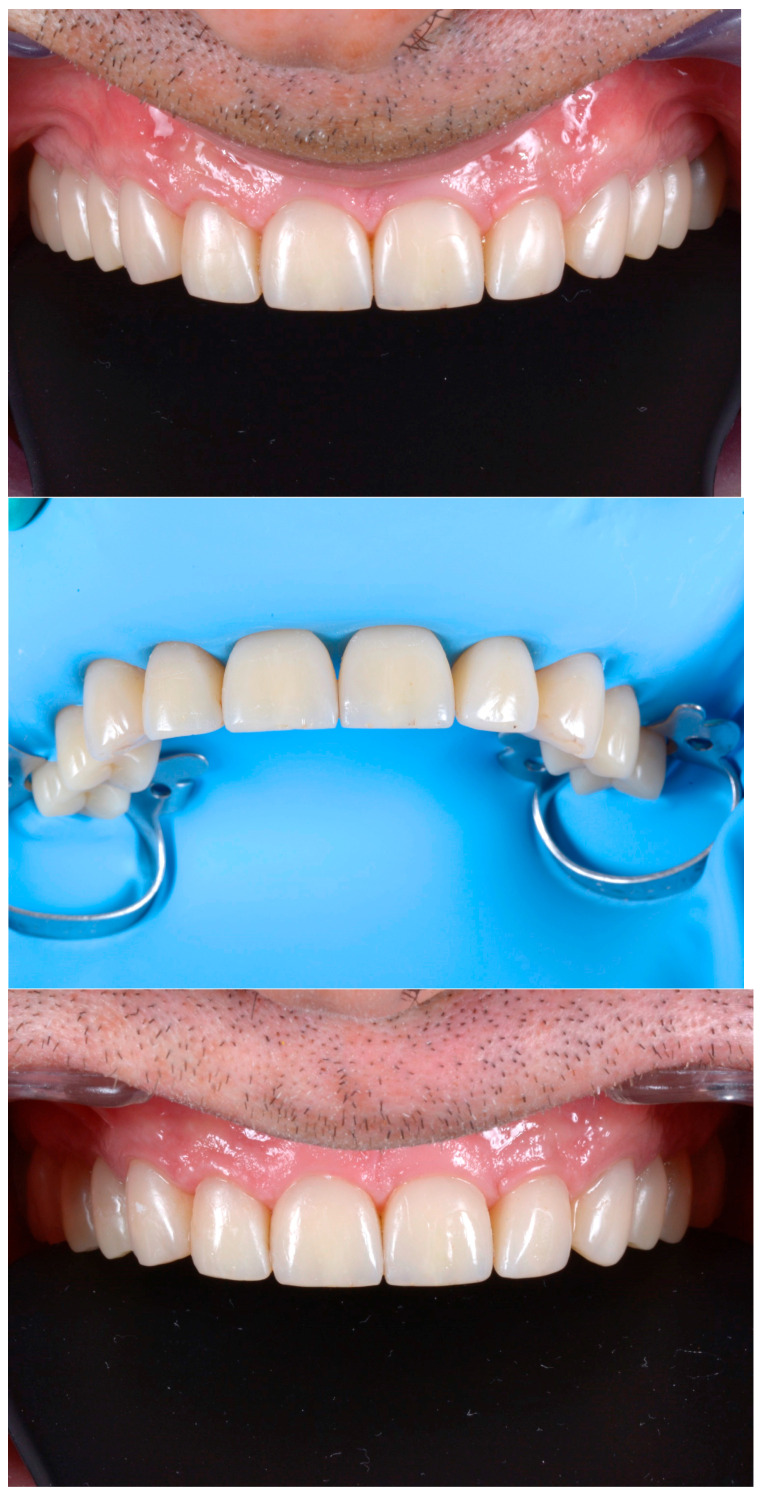
Re-bonding of the incisal edges on teeth: maxillary left central incisor, maxillary left lateral incisor, and maxillary left canine.

At the 6-year follow-up, it was decided to undertake a re-bonding procedure on the marginal edges of teeth maxillary left central incisor, maxillary left lateral incisor, and maxillary left canine because of superficial stains’ infiltration of the composite cutback. The procedure comprised sandblasting, application of an adhesive system (Optibond FL, Kerr, Kloten, Switzerland), and composite application (Filtek Supreme, 3M Company, St. Paul, MN, USA) (Figure 27).

**Figure 28:**
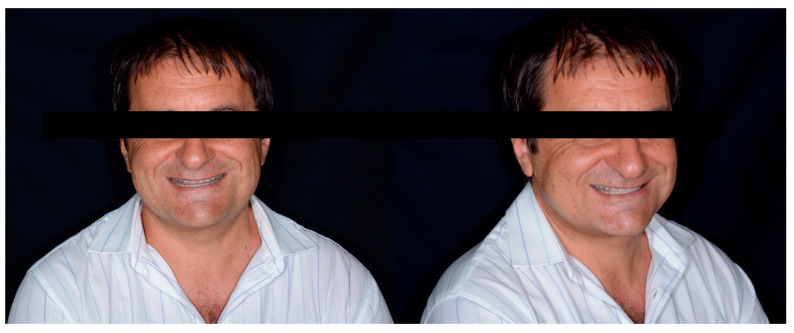
Extra-oral photos at Seven-year follow-up.

**Figure 29:**
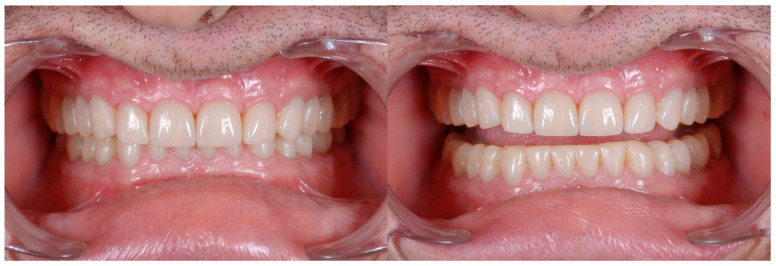
Seven-year follow-up.

Finally, intra and extra oral photos at 7 years of follow-up were acquired (Figure 28 and Figure 29).

**Figure 30:**
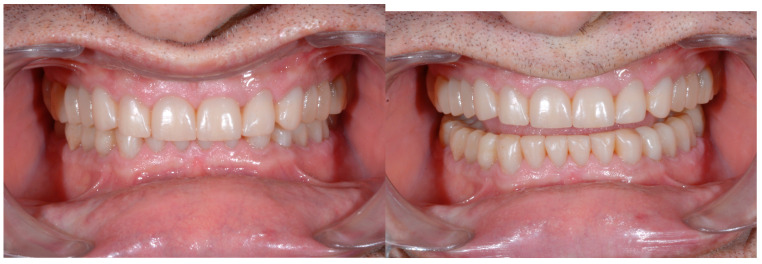
Ten-year follow-up.

**Figure 31:**
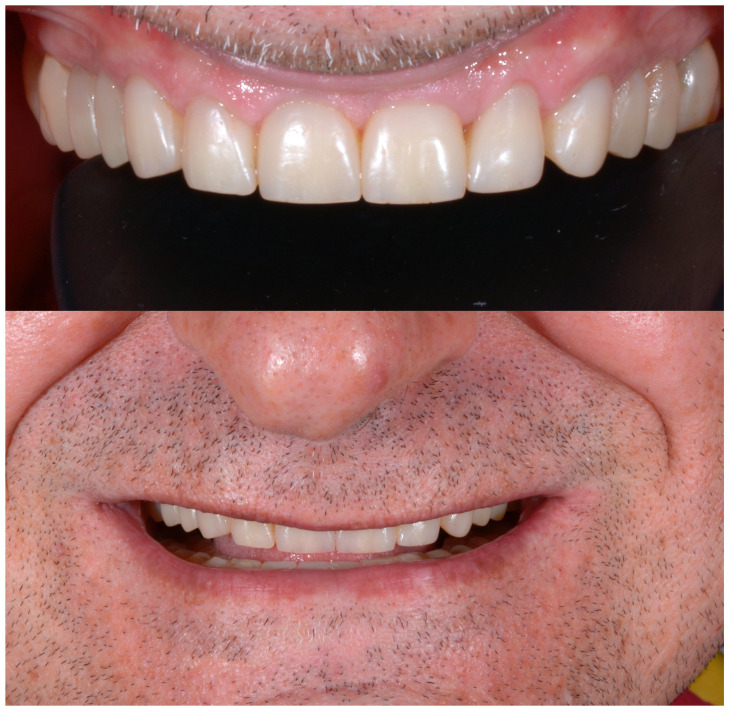
Ten-year follow-up.

**Figure 32:**
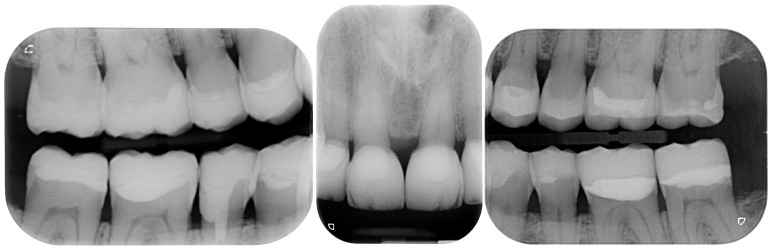
Ten-year follow-up.

A new clinical and radiographic check-up was carried out 10 years after the conclusion of the therapy. The documentation reported here demonstrates the long-term stability of the result of the therapy from an aesthetic, functional, and periodontal point of view (Figure 30, Figure 31 and Figure 32).

## 3. Discussion

Today, thanks to an overall greater awareness and a wider diffusion of clinical knowledge, it is imperative to recognize these diseases at the initial level and propose simplified restorative methods, which involve the minimum sacrifice of healthy tissues.

Removing healthy tooth structure to promote the longevity of restorations may be a contradictory act to achieve repair.

In the authors’ opinion, restorations should instead protect the remaining tooth structures and if they are not thick enough, restorations should be delivered anyway, even if the teeth are weak, since they aim to avoid further degradation of the dentition.

No attempt to reduce healthy tooth structure should be performed, especially if a full-mouth rehabilitation is necessary and an increase in VDO possible.

Patients should be informed very carefully about this shift of paradigm and accept the potential failure of the restorations with the knowledge that the same type of restoration could be repaired/replaced without any damage to the underlying tooth structure.

Since the dentition is reconstructed immediately, this approach is called Speed-Up Therapy, and it substitutes the classic 3 Step Technique in case an esthetic and functional test drive is necessary before delivering the final restorations.

In the 3 Step Technique, the treatment started with provisional restorations in hybrid composite resin, and after 2 weeks the patient is restored with final restorations at the level of the maxillary anterior teeth (palatal veneers).

In Speed-Up Therapy, the VDO is already increased by including anterior contacts using the therapeutic bite, made of provisional composite resin.

Today, thanks to the improvements of the adhesive techniques, cases of erosive tooth wear could be intercepted at earlier stages and restored by adding only what has been lost (additive dentistry).

In the author’s opinion, reconstructive treatment, based mostly on additive dentistry, should be proposed to patients as soon as dentin exposure is present, especially if the causes of the tooth wear are still active.

In the present report, to complete full-mouth rehabilitation, three different techniques were combined: Speed-Up Therapy to stabilize the occlusion of the patient; BOPT to restore the maxillary anterior teeth; the 3 Step Technique to complete the adhesive rehabilitation of the posterior quadrants. The combination of these three different approaches provided the clinician with more opportunities to successfully complete the difficult therapeutic task. The present study is also the first one in the literature evaluating the treatment of extensive erosion damage with a follow-up of 10 years.

Today, the ideal dentistry should be additive and not subtractive.

One of the most interesting aspects concerns the temporary approach realized in one single appointment, without any dental preparation, using a completely additive method; the patient has been placed in the condition to test the project both from an aesthetic and above all functional point of view. A “functional and aesthetic” evaluation was required, based on full-mouth mock-up provisional composite restorations (Speed-Up Therapy—therapeutic bite).

This functional and aesthetic provisional phase was necessary before proceeding towards the extreme bonding procedures previewed.

The choice of the restorative material has fallen to 28 CAD-CAM nano-hybrid composite resin restorations in order to permit eventual simple and economical repairs or modifications directly in the oral cavity.

Moreover, in the present case, we opted for a light surface roughening and removal of the outer sclerotic layer, following evidence suggesting that selective mechanical removal can improve the micromechanical interlocking of adhesives and enhance bond strength. Studies have shown that pretreatment with rotary instruments or air abrasion can significantly increase the bonding potential to eroded dentin by exposing a more receptive substrate for adhesion [35,36]. This approach was adopted to promote the better long-term stability of the adhesive interface, especially in cases with such extensive tissue loss that optimal adhesion was critical for restorative success.

Given the lack of a universally accepted protocol, our decision was based on both literature support and clinical judgment, balancing the need for improved adhesion with the principle of minimal intervention.

Another important aspect to consider in the adhesive protocol is the use of phosphoric acid etching. While traditional total-etch techniques involving phosphoric acid have long been considered the gold standard for enamel bonding, their use on dentin—especially sclerotic or eroded dentin—remains controversial due to the risk of over-etching and compromised hybrid layer formation.

In this case, we opted to use total-etch technique with phosphoric acid followed by the application of primer and bonding adhesive. Another approach supported by the literature, indicates that selective etching provides optimal bonding to enamel without exposing dentin to excessive demineralization [36,37]. Additionally, studies have shown that universal adhesives used in etch-and-rinse or selective-etch mode offer more reliable and durable bonds to enamel compared to self-etch mode alone [38].

This technique allows to maximize adhesion to enamel minimizing technique sensitivity and preserving dentin integrity- an essential consideration incases of advanced tooth wear where dentin exposure is often extensive.

It is also important to emphasize that erosive tooth wear (ETW) exists on a spectrum of severity, and the management approach should be adapted accordingly. The Basic Erosive Wear Examination (BEWE) provides a standardized method to classify the extent of wear and guide treatment decisions. In this case, when the patient first sought treatment, they were already at a moderate-to-severe BEWE score, indicating the need for a restorative approach [6]. Although the rehabilitation was postponed due to the patient’s decision, the extent of the tooth wear at that earlier stage would have still required full-arch adhesive restoration, although potentially with less extensive intervention. Thus, early treatment could have allowed for a more conservative management strategy, emphasizing the importance of timely diagnosis and intervention in patients presenting with progressive ETW.

Probably even in the case of major trauma with eventual complete detachment, frontal restorations could be re-bonded, benefiting from the choice to preserve teeth vitality, avoiding putting inside any posts that, in case of major traumas, may have resulted in the fracture of the root and therefore the irreversible loss of the element.

For the posterior teeth, the complications would also have been present, but less experimental, since several authors have already pushed the limits of adhesive dentistry with adhesive restorations [39].

The overall treatment was very comfortable for the patient and less complicated for the clinician. After 7 and 10 years, a slight loss of occlusal vertical dimension was observed upon visual assessment, but biological, functional, and esthetic success was confirmed. Nevertheless, considering the complexity of the case and the severe dental tissue loss present at baseline, the long-term clinical outcome remains notably satisfactory. The restorations demonstrated good functional and esthetic stability over time, and the minor dimensional change observed is within acceptable limits for a case of this extent [40,41].

## 4. Conclusions

In conclusion, the correct execution of the proposed multidisciplinary approach allowed us to achieve the planned results with excellent accuracy, from both an aesthetic and a functional perspective.

This article illustrates the restorative treatment of a patient with severe erosion through additive adhesive full-mouth rehabilitation, which was successfully achieved thanks to a significant increase in the patient’s vertical dimension of occlusion (VDO). The novelty of this approach lies in the combination of Speed-Up Therapy, BOPT, and the 3 Step Technique, which together constitute a new integrated method for conservative full-mouth rehabilitation.

Furthermore, the successful transformation of the Speed-Up Therapy from temporary to definitive—adapted to the patient’s timing and financial availability—demonstrates the long-term stability of the results over a 10-year follow-up, confirming biological, functional, and aesthetic success.

## Data Availability

The data presented in this study are available on request from the corresponding author.
